# A single-hole spin qubit

**DOI:** 10.1038/s41467-020-17211-7

**Published:** 2020-07-10

**Authors:** N. W. Hendrickx, W. I. L. Lawrie, L. Petit, A. Sammak, G. Scappucci, M. Veldhorst

**Affiliations:** 1 QuTech and Kavli Institute of Nanoscience, Delft University of Technology, P. O. Box 5046, 2600 GA Delft, The Netherlands; 2 QuTech and Netherlands Organisation for Applied Scientific Research (TNO), Stieltjesweg 1, 2628 CK Delft, The Netherlands

**Keywords:** Quantum information, Quantum dots, Qubits

## Abstract

Qubits based on quantum dots have excellent prospects for scalable quantum technology due to their compatibility with standard semiconductor manufacturing. While early research focused on the simpler electron system, recent demonstrations using multi-hole quantum dots illustrated the favourable properties holes can offer for fast and scalable quantum control. Here, we establish a single-hole spin qubit in germanium and demonstrate the integration of single-shot readout and quantum control. We deplete a planar germanium double quantum dot to the last hole, confirmed by radio-frequency reflectrometry charge sensing. To demonstrate the integration of single-shot readout and qubit operation, we show Rabi driving on both qubits. We find remarkable electric control over the qubit resonance frequencies, providing great qubit addressability. Finally, we analyse the spin relaxation time, which we find to exceed one millisecond, setting the benchmark for hole quantum dot qubits. The ability to coherently manipulate a single hole spin underpins the quality of strained germanium and defines an excellent starting point for the construction of quantum hardware.

## Abstract (web summary)

While most results so far in semiconductor spin-based quantum computation use electron spins, devices based on hole spins may have more favourable properties for quantum applications. Here, the authors demonstrate single-shot readout and coherent control of a qubit made from a single hole spin.

## Introduction

Group-IV semiconductor spin qubits^1^ are promising candidates to form the main building block of a quantum computer owing to their high potential for scalability towards large 2D-arrays^2–5^ and the abundance of net-zero nuclear spin isotopes for long quantum coherence^6,7^. Over the past decade, all prerequisites for quantum computation were demonstrated on electron spin qubits in silicon, such as single-shot readout of a single electron^8^, high-fidelity single-qubit gates^9,10^ and the operation of a two-qubit gate^11–14^. However, hole spins may offer several advantages^15,16^, such as a strong spin-orbit coupling (SOC) and a large excited state energy. Early research demonstrated the feasibility of using the SOC for all-electric driving^17,18^, but these experiments were limited by nuclear spins and the coherent driving of a single-hole spin remained an open challenge. More recently, hole spins in group-IV materials have gained attention as a platform for quantum information processing^19–22^. In particular, hole states in germanium can provide a high degree of quantum dot tunability^23–25^, fast and all-electrical driving^20,21^ and Ohmic contacts to superconductors for hybrids^26,27^. These experiments culminated in the recent demonstration of full two-qubit logic^21^. Although hole spins have been read out in single-shot mode using the Elzerman technique^28^, these experiments require magnetic fields impractical for hole qubit operation owing to the strongly anisotropic *g*-factor of hole spins in germanium^29^. Pauli spin blockade (PSB) readout allows for spin readout independent of the Zeeman splitting of the qubit, leveraging the large excited state energy purely defined by the orbital energy for holes in germanium. Furthermore, achieving these assets on a single-hole spin demonstrates full control over the materials system and allows to tune the quantum dot occupancy at will, optimising the different qubit properties. Moreover, the ability to study a platform at the single-particle level would provide great insight into its physical nature, crucial for holes that originate from a more-complicated band structure than electrons^30,31^.

In this work, we make this step and demonstrate single-shot readout and operation of a single-hole spin qubit. We grow undoped strained germanium quantum wells^32^ and fabricate devices using standard manufacturing techniques^2^. The high mobility and low effective mass^33^ allow us to define quantum dots of relatively large size, alleviating the restraints on fabrication. We deplete the quantum dots to their last hole, confirmed by charge sensing using a nearby single-hole transistor (SHT). The use of radio-frequency (RF) reflectometry^34–36^ enables a good discrimination of the charge state while maintaining a high measurement bandwidth to allow for fast spin readout. We make use of PSB to perform the spin-to-charge conversion^37^, maximally taking advantage of the large excited state energy splitting of *E*_ST_ = 0.85 meV and obtain single-shot spin readout. Finally, we demonstrate the integration of readout and qubit operation by performing all-electrically driven Rabi rotations on both qubits. Studying the control of a single-hole qubit, we find a remarkably strong dependence of the resonance frequency on electric field and show a tunability of almost 1 GHz using only small electric potential variations.

## Results

### Single-hole quantum dot and PSB

**Fig. 1:**
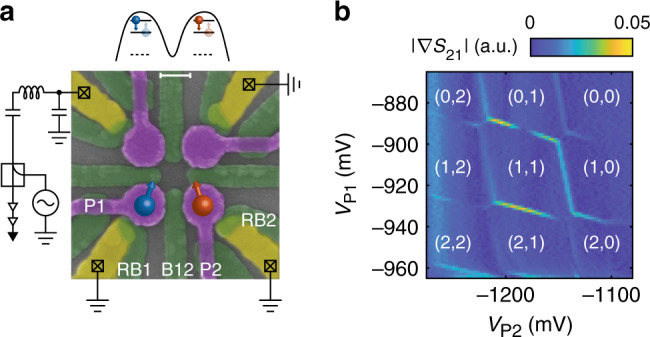
Fabrication and operation of a planar germanium double quantum dot. **a** False-coloured scanning electron microscope image of the quadruple quantum dot device. Ohmic contacts are indicated in yellow, a first layer of electrostatic barrier gates is indicated in green and the second layer of plunger gates is coloured in purple (for details, see Methods). The scale bar corresponds to 100 nm. We use the double quantum dot in the top channel as a single-hole transistor (SHT) to sense changes in the charge occupation of the quantum dots formed under plunger gates P1 and P2. A schematic illustration of the electrostatic potential defining the two single-hole quantum dots is depicted above the figure. The charge sensor impedance is measured using reflectometry on a resonant circuit consisting of a superconducting resonator and the parasitic device capacitance. Barrier gates RB1 and RB2 can be used to control the tunnel rate of each quantum dot to its respective reservoir and gate B12 controls the interdot tunnel coupling. **b** Charge stability diagram of the double quantum dot system, where depletion of both quantum dots up to the last hole can be observed.

A false-coloured SEM picture of the quantum dot device is depicted in Fig. 1a. The device consists of a quadruple quantum dot system in a two-by-two array^2^. We tune the top two quantum dots into the many-hole regime, such that they can be operated as a SHT. In order to perform high-bandwidth measurements of the sensor impedance, we make use of RF-reflectometry, where the SHT is part of a resonant LCR-circuit further consisting of an off-chip superconducting resonator together with the parasitic device capacitance. We apply a microwave signal to the tank circuit and measure the amplitude of the signal reflected by the LCR-circuit (see Fig. 1a). The amplitude of the reflected signal ∣*S*_21_∣ depends on the matching of the tank circuit impedance with the measurement setup and is therefore modulated by a change in the charge sensor impedance caused by the movement of a nearby charge.

**Fig. 2:**
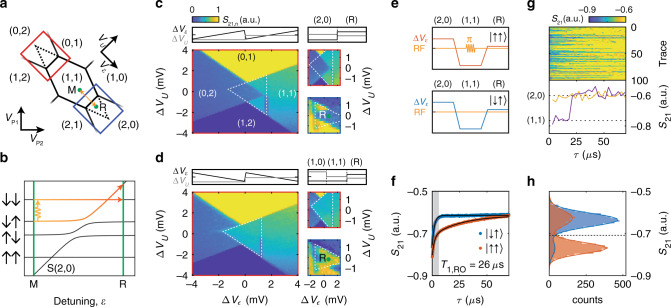
Single-shot spin readout of a single hole. **a** Schematic of a typical hole charge stability diagram with both possible regions of readout indicated in blue and red. The typical manipulation (M) and readout (R) points are indicated in green. **b** Two-hole energy diagram, with the five lowest lying energy states around the (1,1)-S(2,0) anticrossing. **c** Colour map of the normalised sensor response (normalisation in Methods) as a function of the applied gate voltages *V*_*U*_ and *V*_*ϵ*_. In the large panel, we linearly sweep *V*_*ϵ*_ and step *V*_*U*_, as indicated in the inset above the figure. The smaller panels on the right show the same effect for the (1,1)-(0,2) anticrossing (top, red box in **a**), and the (1,1)-(2,0) anticrossing (bottom, blue box in **a**), now using a two-level voltage pulse (details in Methods). **d** Similar colour map as in **c**, but with a reversed sweeping direction from (1,1) to the (0,2) region. The triangular spin blockade window is indicated by the dashed white line. The smaller panels on the right again demonstrate the same effect for both the (1,1)-(0,2) (top) as well as the (1,1)-(2,0) (bottom) anti-crossings, by first loading a random spin in one of the dots (details in Methods). **e** Schematic illustration of the three-level pulses used in **f**–**h**, indicating the detuning voltage *ΔV*_*ϵ*_ in blue and red, and the RF-pulses in orange. **f** The averaged charge sensor response as a function of measurement time *τ* at R for $\left|\uparrow \uparrow \right\rangle$ initialisation (red) and $\left|\downarrow \uparrow \right\rangle$ initialisation (blue). The grey-shaded area indicates the integration window for the threshold detection. **g** A sample of 100 single-shot traces (top), averaged for 3 μs per data point, with *τ* = 0 the start of the readout phase. The bottom panel shows two single traces, where the purple (yellow) trace corresponds to the readout of a blocked (not blocked) spin state. Dashed lines correspond to the sensor signal for the different charge states. **h** Histogram of 5000 single-shot traces, integrating the signal for 5.5 μs as indicated in **f**. The blue (red) histogram corresponds to an initialisation in the $\left|\downarrow \uparrow \right\rangle$ ($\left|\uparrow \uparrow \right\rangle$) state. The dashed line corresponds to the optimised threshold for readout.

We make use of the RF sensor to map out the charge stability diagram of the double quantum dot system defined by plunger gates P1 and P2. The tunnel coupling of the quantum dots to their reservoirs, as well as the interdot tunnel coupling can be tuned by gates RB1, RB2 and B12, respectively. Next, we tune the device to the single-hole regime for both quantum dots (Fig. 1b and Supplementary Fig. 1), where (*N*_1_, *N*_2_) indicates the charge occupation, with *N*_1_ (*N*_2_) the hole number in the dot under P1 (P2). In our previous work^2^, we further detail that we can deplete all four quantum dots in this device down to their last hole. In order to perform readout of the spin states, we make use of PSB, which is expected to be observed both at the (1,1)-(0,2) and (1,1)-(2,0) charge transitions. We define the virtual gates^38^ detuning *V*_*ϵ*_ and energy *V*_*U*_ (see Fig. 2a and Methods) and sweep across the (1,1)-(2,0) and (1,1)-(0,2) transitions in this gate space. As a result of its triplet character, the $\left|\downarrow \downarrow \right\rangle$ state has a negligible coupling to the S(2,0) or S(0,2) singlet charge states (Fig. 2b). When pulsing across the (1,1)-S(2,0) or (1,1)-S(0,2) anti-crossings, PSB prevents charge movement when the system is in the $\left|\downarrow \downarrow \right\rangle$ ground state. However, when the system resides in the singlet-like lower antiparallel spin state (in this case $\left|\downarrow \uparrow \right\rangle$, with Q2 being the qubit with lower Zeeman energy), charge movement to a doubly occupied quantum dot state is possible, therefore leaving the system in a (0,2) or (2,0) charge state. This results in a spin-to-charge conversion, which in turn can be picked up in the reflectometry signal from the SHT.

Indeed, we find that by sweeping the detuning voltage across the interdot transition from the (1,1) to the (0,2) charge region (Fig. 2d), tunnelling is blocked up to the reservoir transitions (indicated in white) when the system is initialised in the $\left|\downarrow \downarrow \right\rangle$ state. In this case, we rely on the fast diabatic return sweep combined with fast spin relaxation compared with the sweep rate to prepare the system in the blocking $\left|\downarrow \downarrow \right\rangle$ state. When we inverse the sweeping direction, the system remains in the (0,2) charge states at the same values of *V*_*ϵ*_ and *V*_*U*_ (Fig. 2c). After optimising the different tunnel rates in the device, we confirm the PSB at both the (1,1)-(2,0) and (1,1)-(0,2) anti-crossings by loading a random spin before performing the readout, thereby not relying on a relaxation process for the initialisation (small panels of Fig. 2c, d). The diamond-shaped window of differential signal allows for a singlet/triplet readout of the system spin state and we select readout point R (see Supplementary Fig. 2). We note that the interdot transition line is shifted slightly towards positive detuning with respect to the reservoir transition lines. This is the direct result of a small voltage offset present across the device Ohmics, resulting in the unusual diamond-shaped spin readout window, but not limiting the readout. As holes in germanium do not have any valley states, the T(2,0) state is expected to be defined by the next quantum dot orbital. By increasing the bias voltage across the two quantum dots, we shift the interdot transition line further. At large enough bias, the PSB window is capped as a result of the T(2,0) state becoming available in energy, and from this we extract an excited state energy of *E*_ST_ = 0.85 meV, using a lever arm of *α*_*ϵ*_ = 0.21 as extracted from polarisation line measurements (Supplementary Fig. 3).

### Qubit operation

To coherently control the qubits, we implement a three-level voltage pulsing scheme (Fig. 2e) and operate at an external magnetic field of *B* = 0.67 T. We initialise the system by pulsing deep into the (2,0) region (*α*_*ϵ*_*V*_*ϵ*_ > *E*_ST_), where the spins quickly relax into the (2,0) singlet state. Next, we ramp adiabatically into the (1,1) region, preparing the system into the $\left|\downarrow \uparrow \right\rangle$ state. At this point (M), we perform the qubit operations by applying microwave pulses to gate P1, taking advantage of the SOC-mediated electrically driven electron spin resonance. Rotating Q1 (Q2) will bring the system into the $\left|\uparrow \uparrow \right\rangle$ ($\left|\downarrow \downarrow \right\rangle$) state. Finally, the spin-state is read out by pulsing adiabatically into the readout window. Only the $\left|\downarrow \uparrow \right\rangle$ state will allow a direct tunnelling into the (2,0) charge state, whereas tunnelling is blocked for all other states owing to PSB. Fig. 2f displays the charge sensor signal throughout the readout period, both for a $\left|\downarrow \uparrow \right\rangle$ initialisation (blue) as well as a $\left|\uparrow \uparrow \right\rangle$ initialisation (red) by applying a *π*-pulse to Q1. When no pulse is applied and the system is prepared in the $\left|\downarrow \uparrow \right\rangle$ state, a fast transition into the (1,1)-charge state, corresponding to a sensor signal of *S*_21_ ≈ −0.6 can be observed. The remaining decay (*T*_decay_ = 2 μs) in this case can be attributed to the response of the SHT-signal to the voltage pulses on the gates. However, when the system is prepared in the $\left|\uparrow \uparrow \right\rangle$ state, a significantly slower relaxation into the (1,1) state is observed, owing to the spin blockade combined with the slow T_+_(1,1)-S(2,0) relaxation. By fitting a double exponential decay, accounting for the SHT response, we extract a spin relaxation at the readout point of *T*_1,RO_ = 26 μs. A sample of 100 single-shot traces is plotted in Fig. 2g, together with two individual traces using a post-processing integration time of 3 μs. A clear distinction of the (1,1) and (2,0) charge states can be observed from the sensor response. To determine the spin state of the qubits, we perform a threshold detection of the single-shot signal integrated from *τ*_0_ = 1.0 μs to *τ*_meas_ = 6.0 μs for maximised visibility, discarding the initial stabilisation of the SHT and optimising between the charge discrimination and spin relaxation. A histogram of 5000 single-shot events illustrates the clear distinction between the singlet (*S*_21_ > −0.72) and the triplet (*S*_21_ < −0.72) spin-state readout (Fig. 2h). We find a spin readout visibility of *v* = 56% as obtained from the difference in spin-up fraction between the two prepared states. A large part of this reduced visibility is caused by relaxation of the blocked triplet state during the measurement, expected to amount to a signal reduction of ${P}_{{\rm{relax}}}=1-{e}^{-{\tau }_{{\rm{meas}}}/{T}_{1,{\rm{RO}}}}=0.21$. This gives good prospects for increasing the readout fidelity by optimising the spin relaxation, for instance, by optimising the reservoir tunnel rates and moving to latched PSB readout mechanisms^39,40^. Alternatively, by using high-Q on-chip resonators^41^ the signal-to-noise ratio could be significantly improved, thereby lowering the required integration time and reducing the effective relaxation. The remaining triplet fraction of 0.11 that can be observed for the readout of the $\left|\downarrow \uparrow \right\rangle$ state could be attributed to an anadiabaticity of the pulsing or a small coupling between the T(1,1) and S(2,0) states owing to the SOC. This could be mitigated by further optimising the readout pulse sequence.

**Fig. 3:**
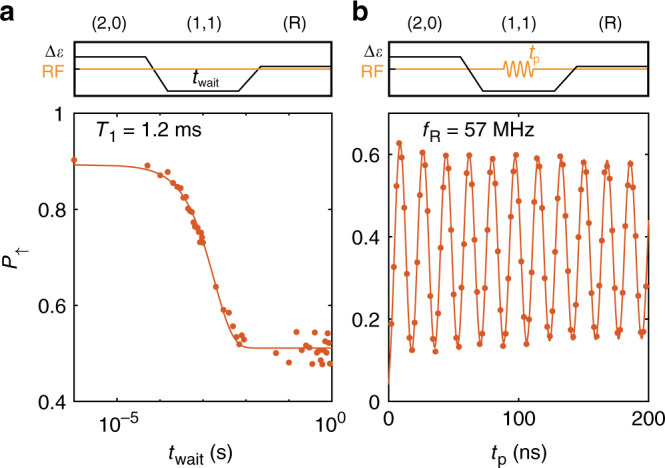
Spin relaxation and coherent driving of a single hole. **a** The system is initialised in the $\left|\downarrow \uparrow \right\rangle$ state after which the qubits idle at the measurement point. The spin-up fraction *P*_*↑*_ of Q2 is measured as a function of waiting time *t*_wait_ and shows a typical *T*_1_-decay with a relaxation time of *T*_1_ = 1.2 ms. **b** Driving of the single-hole qubit Q2 shows coherent oscillations in *P*_*↑*_ as a function of the microwave pulse length *t*_p_. The coherent operation of Q1 is shown in Supplementary Fig. 4.

Now, we probe the single spin relaxation time by initialising the system in the $\left|\downarrow \uparrow \right\rangle$ state and letting the system evolve at a detuning voltage *ΔV*_*ϵ*_ = −7 mV from the (1,1)-(2,0) anticrossing. Fig. 3a shows the spin-up fraction as a function of the waiting time *t*_wait_, from which a single spin relaxation time of *T*_1,Q2_ = 1.2 ms can be extracted. This is substantially longer than reported before in planar germanium heterostructures^21^, most likely as a result of the more isolated single-hole spins as compared with the transport measurements with high reservoir couplings, and is also longer than all relevant time scales for qubit operation. Moreover, this relaxation time compares favourably to results obtained for holes in Ge nanowires^42^, Ge hutwires^28^ and other hole spins^43,44^.

**Fig. 4:**
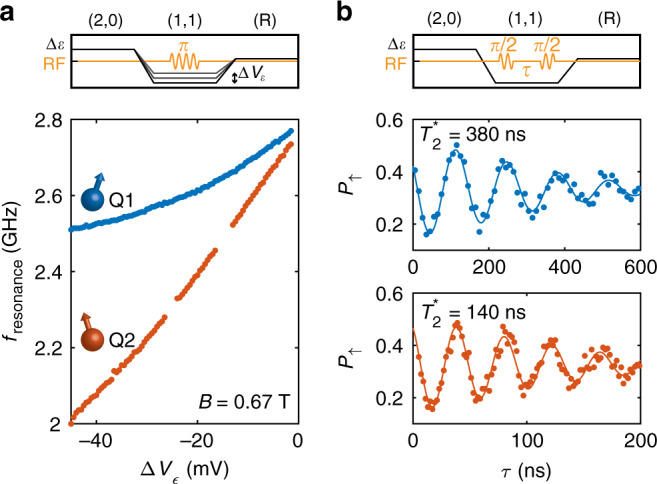
Electric g-factor modulation and phase coherence of the qubit resonances. **a** The resonance frequency of both qubits shows a strong modulation as a function of the detuning voltage *ΔV*_*ϵ*_. **b** We perform a Ramsey experiment on both qubits to probe the phase coherence times, with ${T}_{2,Q1}^{* }=380$ ns and ${T}_{2,Q2}^{* }=140$ ns. The comparatively short phase coherence can be attributed to the strong dependence of *f*_resonance_ to electric fields, coupling charge noise to the spin state, leading to increased decoherence.

To demonstrate coherent control of a single hole, we modulate the length of the driving microwave pulse and measure the spin-up fraction (Fig. 3b). A clear sinusoidal Rabi oscillation can be observed on Q2, with a Rabi frequency of *f*_*R*_ = 57 MHz (coherent operation of Q1 in Supplementary Fig. 4). We probe the phase coherence of both qubits by performing a Ramsey sequence in which we apply two *π*/2-pulses, separated by a time *τ* in which we let the qubit freely evolve and precess at a frequency offset of *Δf* = 7.4 MHz and *Δf* = 23.7 MHz respectively. In Fig. 4b the Ramsey decay for Q1 and Q2 are plotted and we extract coherence times of ${T}_{2,Q1}^{* }=380$ ns and ${T}_{2,Q2}^{* }=140$ ns. These coherence times are of comparable order, but slightly lower than previously reported numbers in the same heterostructere for a many-hole quantum dot^21^. In order to explain the origin of this, we measure the resonance frequency of both qubits as a function of the detuning voltage *ΔV*_*ϵ*_. We find a very strong dependence of the resonance frequency of both qubits on the detuning voltage over the entire range of voltages measured, with the *g*-factor varying between *g*_Q1_ = 0.27−0.3 and *g*_Q2_ = 0.21−0.29. This strong electric field dependence of the resonance frequency will increase the coupling of charge noise to the qubit spin states, which in turn will reduce phase coherence^21^. The ratio in local slopes of the resonance frequency *δf*_Q2_/*δf*_Q1_ = 2 is similar to the ratio in phase coherence of both qubits ${T}_{2,Q1}^{* }/{T}_{2,Q2}^{* }=2.5$, consistent with charge noise limited coherence. The strong modulation of the qubit resonance frequency by electric field could be explained from the strong SOC present^45,46^. This is further supported by the Rabi frequency changing as a function of detuning voltage (see Supplementary Fig. 5), as is predicted to be a result of the strong SOC^45,46^. We attribute the slightly different resonance frequency of Q1 and Q2 to an asymmetry in the potential landscape of the two dots. Although the strong *g*-factor modulation seems mainly a cause of decoherence in this case, careful optimisation of the electric field landscape could render a situation in which the qubit Zeeman splitting is well controllable, while maintaining a zero local slope for high coherence^46^.

## Discussion

The demonstration that single-hole spins can be coherently controlled and read out in single-shot mode, together with the spin relaxation times *T*_1_ > 1 ms, defines planar germanium as a mature quantum platform. These aspects are demonstrated on a two-dimensional quantum dot array, further highlighting the advancement of germanium quantum dots. Moreover, controlling a single-hole spin represents an important step toward reproducible quantum hardware for scalable quantum information processing.

## Methods

### Fabrication process

We grow strained Ge/SiGe heterostructures in an Epsilon 2000 (ASMI) reduced-pressure chemical vapour deposition reactor on a 100 mm n-type Si(001) substrate. The growth sequence comprises a 1.6-μm-thick relaxed Ge layer; a 1 μm-thick step-graded Si_1−*x*_Ge_*x*_ layer with final Ge composition *x* = 0.8; a 500-nm-thick strain-relaxed Si_0.2_Ge_0.8_ buffer layer; a 16-nm-thick strained Ge quantum well; a 22-nm-thick strain-relaxed Si_0.2_Ge_0.8_ barrier; a sacrificial Si cap layer < 2 nm thick. Further details on the heterostructure are discussed in ref. ^32^. Ohmic contacts are defined by electron beam lithography, electron beam evaporation and lift-off of a 30-nm-thick Al layer. Electrostatic gates consist of a Ti/Pd layer with a thickness of 20 and 40 nm, respectively, for the barrier and plunger gate layer. Both layers are separated from the substrate and each other by 10 nm of ALD-grown Al_2_O_3_.

### Experimental setup

We use a Bluefors dry dilution refrigerator with a base temperature of *T*_bath_ ≈ 20 mK to perform the measurements. Battery-powered voltage sources are used to supply DC-voltages on the gates. In addition, AC-voltages generated by a Tektronix AWG5014C arbitrary waveform generator can be supplied to the gates through a bias-tee with a cutoff frequency of  ≈10 Hz. Similarly, we can also apply a microwave signal generated by a Keysight PSG8267D vector source to gate P1 for qubit driving. Driving both qubits at the same power on gate P2, we observe significantly slower Rabi oscillations in Q1. From this we assume Q1 to be located under P1, and thus Q2 under P2, in correspondence with the trend in Rabi frequencies observed in a previous work^21^.

We use an in-house built RF generator to supply the reflectometry signal. The signal is attenuated by 84 dB and applied to one of the sensor Ohmics via a Mini-Circuits ZEDC-15-2B directional coupler. The reflected signal is amplified by a Caltech CIRLF3 SiGe-amplifier at the 4 K-stage of our fridge and an in-house built RF-amplifier at room temperature, and finally IQ-demodulated to give a measure of *S*_21_.

### Measurement details

The large panels of Fig. 2c, d are measured by continuously sweeping *ε* and stepping *U*, while measuring the sensor response. The smaller panels in Fig. 2c show the sensor response after applying a two-level voltage pulse to load the (2,0) or (0,2) charge configuration and vary the readout point across the map. The signal is then integrated for 10 μs at each pixel. The smaller panels in Fig. 2d show the sensor response after applying a three-level voltage pulse to first randomly load a spin in the second dot by pulsing across the (1,0)-(1,1) reservoir transition. Next, we pulse across the (1,1)-(0,2) or (1,1)-(2,0) interdot transition to perform the spin readout. The colour scale of the signal in Fig. 2c left and Fig. 2d left panels is normalised by *S*_21,*n*_ = 10*S*_21_ + 3.5. The top right panel in Fig. 2c is normalised by *S*_21,*n*_ = 12.5S_21_ + 5. The bottom right panel in Fig. 2c is normalised by *S*_21,*n*_ = 20S_21_ + 17.2. The top right panel in Fig. 2d is normalised by *S*_21,*n*_ = S_21,*n*_ = 12.5S_21_ + 5.375. The bottom right panel in Fig. 2d is normalised by *S*_21,*n*_ = 25*S*_21_ + 20.75, with *S*_21,*n*_ the normalised sensor signal as plotted in Fig. 2 and *S*_21,*n*_ the demodulated sensor signal strength in mV.

For the measurements in Fig. 2f–h and Fig. 3 and 4, typical adiabatic ramp times of *T*_ramp_ ≈ 1 μs are used.

### Virtual gates

In order to allow independent control over the detuning and energy of the quantum dots more easily, we define the virtual gate axes of *V*_*ε*_ = *V*_P2_ − 0.5 *V*_P1_ and *V*_*U*_ = 0.5 *V*_P2_ + *V*_P1_.

## Supplementary information

Supplementary Information

## Data Availability

All data underlying this study are available from the 4TU ResearchData repository at 10.4121/uuid:5d77f9d7-bfa5-4a85-a147-82dd948d32d4.
