# Supplementary material for: A single-hole spin qubit
Source: Nat Commun. 2020 Jul 10;11:3478. doi: 10.1038/s41467-020-17211-7 (PMC7351715; doi:10.1038/s41467-020-17211-7)
Supplement: Supplementary file 1 — Supplementary Information [file 41467_2020_17211_MOESM1_ESM.pdf]

Supplementary Information: A single-hole spin qubit

N.W. Hendrickx,[1,*] W.I.L. Lawrie,[1] L. Petit,[1] A. Sammak,[2] G. Scappucci,[1] and M. Veldhorst[1,†]

[1]*QuTech and Kavli Institute of Nanoscience, Delft University of Technology, P.O. Box 5046, 2600 GA Delft, The Netherlands*
[2]*QuTech and Netherlands Organisation for Applied Scientific Research (TNO), Stieltjesweg 1, 2628 CK Delft, The Netherlands*
(Dated: May 18, 2020)

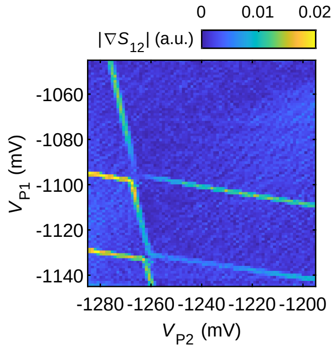

Supplementary Fig. 1. **Depletion of a hole double quantum dot in germanium.** Colour map of the sensor signal as a function of the voltages on plunger gates P1 and P2. No extra addition lines can be observed beyond $V_{\mathrm{P1}} \approx -1100$ and $V_{\mathrm{P2}} \approx -1260$, indicating the double quantum dot is fully depleted. This is the same anti-crossing as observed in Fig. 1b of the main text and the slight decrease of plunger gate voltages can be attributed to a time-dependent hysteretic drift as a result of extensive gate voltage sweeping.

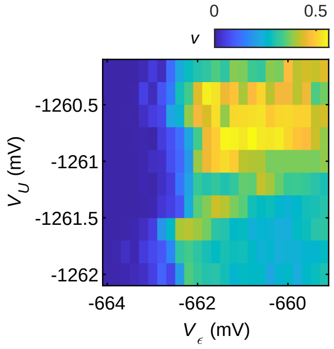

Supplementary Fig. 2. **Optimisation of the readout-point.** Colour map of the visibility of the readout v, as defined by $v = P_{\mathrm{blocked},\pi} - P_{\mathrm{blocked},0}$, with $P_{\mathrm{blocked},\pi}$ being the probability of measuring a blocked signal after applying a resonant π-pulse to Q1 and $P_{\mathrm{blocked},0}$ the probability of measuring a blocked signal without applying any microwave pulses. A clear optimal spot for readout can be observed at $V_U = -1260.7$ mV, $V_\epsilon = -661.0$ mV.

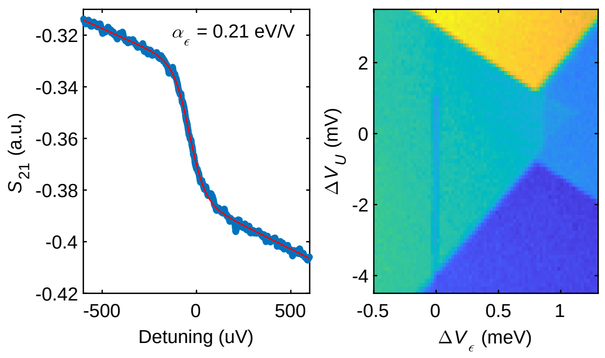

Supplementary Fig. 3. **Lever arm and excited state energy of the quantum dot. a** Polarisation line measurement of the (1,1)-(0,2) anticrossing. We fit the thermally limited polarisation line to a model including cross-talk to the charge sensor and the effect of the charge state on the sensor sensitivity [1]. Assuming a hole temperature of 100 mK as measured previously [2], we find a lever arm of $\alpha_\epsilon = 0.21$ eV/V, in good agreement with results obtained on similar devices. **b** We measure the excited state energy by applying a DC bias across the quantum dot ohmics, shifting the anti-crossing towards the negative detuning voltage. For large enough bias, the readout window is capped off, as a result of the excited state becoming available in energy. From this we deduce an excited state splitting of $E_{ST} = 0.85$ meV.

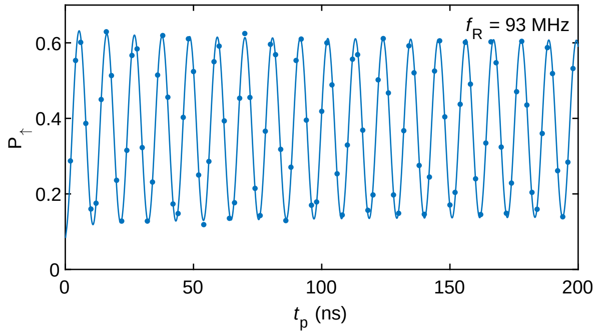

Supplementary Fig. 4. **Coherent operation of Q1.** We rotate Q1 by applying a resonant microwave pulse to gate P1 and observe fast Rabi oscillations, with a frequency of $f_R = 93$ MHz.

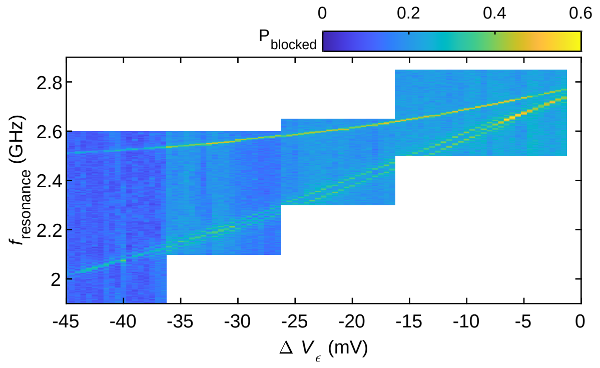

Supplementary Fig. 5. **Detuning dependence of the resonance frequency of Q1 and Q2.** Colour map indicating a blocked state fraction P_{blocked} as a function of the detuning voltage ΔV_ϵ of the manipulation point. We apply a microwave pulse to gate P2 with a duration of $t_p = 105$ ns, corresponding to an approximate 3π-pulse on Q2 and a π-pulse on Q1 at $\Delta V_\epsilon = -5$ mV. The resonance line corresponding to Q2, can be observed to split and recombine throughout the map, as a direct result of the Rabi frequency changing resulting in rotations exceeding a 3π-pulse.

[1] DiCarlo, L. *et al.* Differential Charge Sensing and Charge Delocalization in a Tunable Double Quantum Dot. *Phys. Rev. Lett.* **92**, 226801 (2004).
[2] Petit, L. *et al.* Spin Lifetime and Charge Noise in Hot Silicon Quantum Dot Qubits. *Phys. Rev. Lett.* **121**, 076801 (2018).
